# Health-Related Internet Usage and Design Feature Preference for E-Mental Health Programs Among Men and Women

**DOI:** 10.2196/11224

**Published:** 2019-03-18

**Authors:** Rachel Smail-Crevier, Gabrielle Powers, Chelsea Noel, JianLi Wang

**Affiliations:** 1 Work & Mental Health Research Unit The Royal's Institute of Mental Health Research University of Ottawa Ottawa, ON Canada; 2 School of Epidemiology and Public Health Faculty of Medicine University of Ottawa Ottawa, ON Canada

**Keywords:** occupational health, workplace, mental health, preventive health program, depression, internet

## Abstract

**Background:**

Major depressive episodes (MDEs) are prevalent in the workplace and affect workers’ health and productivity. Therefore, there is a pressing need for innovation in the prevention of MDEs in the workplace. Electronic mental (e-mental) health programs are a cost-effective approach toward the self-management of stress and emotional issues. E-mental health dropout rate, MDE prevalence, and symptoms greatly vary by sex and age. Thus, the development and implementation of e-mental health programs for the prevention of MDEs need to be examined through a sex and age lens to enhance program use and effectiveness.

**Objective:**

This study aimed to examine design feature preferences based on sex and age for an e-mental health program targeted toward depression prevention.

**Methods:**

Household residents across Canada were contacted using the random digit dialing method. 500 women and 511 men who were 18 years and older and who were at high risk of having MDEs were interviewed. Internet use was assessed using questions from the 2012 Canadian Internet Use Survey conducted by Statistics Canada, and preferred design features of e-mental health program questions were developed by the BroMatters team members. The proportions of likely use of specific features of e-mental health programs in women were estimated and compared with those in men using chi-square tests. The comparisons were made overall and by age groups.

**Results:**

Men (181/511, 35.4%) and women (211/500, 42.2%) differed significantly in their likelihood of using an e-mental health program. Compared with men (307/489, 62.8%), women (408/479, 85.2%) were more likely to use the internet for medical or health-related information. Women were more likely to use the following design features: practices and exercises to help reduce symptoms of stress and depression (350/500, 70.7%), a self-help interactive program that provides information about stress and work problems (302/500, 61.8%), the ability to ask questions and receive answers from mental health professionals via email or text message (294/500, 59.9%), and to receive printed materials by mail (215/500, 43.4%). Men preferred to receive information in a video game format (156/511, 30.7%). Younger men (46/73, 63%) and younger women (49/60, 81%) were more likely to access a program through a mobile phone or an app, and younger men preferred having access to information in a video game format.

**Conclusions:**

Factors such as sex and age influenced design feature preferences for an e-mental health program. Working women who are at high risk for MDEs preferred interactive programs incorporating practice and exercise for reducing stress, quality information about work stress, and some guidance from professionals. This suggests that sex and age should be taken into account when designing e-mental health programs to meet the needs of individuals seeking help via Web-based mental health programs and to enhance their use.

## Introduction

Major depressive episodes (MDEs) are prevalent in the workplace and affect workers’ health and productivity. In the United States, workers with depression cost an estimated US $44.01 billion per year in lost productivity [1]. One of the severe consequences of having an MDE is suicide [2]. Given the significant disease burden, there is a pressing need for innovation in the prevention of MDEs in the workplace. A number of approaches have been proposed and investigated to help workers. Studies in the workplace have focused on providing treatment services to employees with depression [3], including cognitive behavioral therapy (CBT) conducted by professionals, diagnosis of depression, enhanced primary care delivery, treatment by medical doctors, integrated care management, and worksite stress reduction [3-6]. At the population level, despite the significant increase in antidepressant use in the past two decades [7], there has been no measurable change in the prevalence of MDEs in different countries [8-11]. A recent systematic review concluded that there is insufficient evidence to determine which individual-based interventions improve depression management in the workplace [12]. In contrast, another recent meta-analysis concluded that secondary interventions involving CBT-based stress management and tertiary interventions with specific focus on work such as exposure therapy and CBT-based and problem-focused return-to-work programs improved both symptoms of common mental disorders and occupational outcomes [13]. To date, most workplace responses to the burden of mental health problems have been reactive, with the implementation of workplace interventions only being considered once a worker is symptomatic or even on sick leave [14]. Evidence suggests that various mental health problems can be prevented [15]. As a result, policy makers and researchers have begun to consider strategies aimed at early prevention [15,16], for example, identifying and treating asymptomatic persons who have already developed risk factors but in whom the condition has not become clinically apparent (high-risk individuals). From a public health perspective, universal interventions are attractive for their ability to reach more working adults and because they have the ability to reach specified groups and individuals without screening them, an exercise that has shown to be costly [17,18]. In terms of depression prevention, one cost-effective strategy for helping those who are at high risk of depression is an electronic mental (e-mental) health program [19,20].

In the past decade, there has been considerable interest in the delivery of e-mental health programs, that is, delivering mental health services through the internet or mobile apps. Web-based interventions and mobile apps can be utilized for self-help or for treatment purposes [21]. E-mental health programs are either guided or unguided and draw on the internet and other media, including video, phone, and apps for mobile phones and tablets [22], and generally address 4 areas of mental health service delivery: (1) information provision; (2) screening, assessment, and monitoring; (3) intervention; and (4) social support (sometimes concurrently) [19]. The wide use of internet and mobile devices has laid a strong foundation for eHealth development. In 2011, 83% of Canadians reported using Web-based services [23], and in 2014, 99% of young Canadians reported having access to the internet outside of school [24]. The number of individuals using the internet to search for medical or health-related information increased from 57.9% in 2005 to 69.9% in 2009 [25]. Over 60% of internet users use wireless handheld devices to access the internet [23]. With such a large portion of Canada’s population online and with many young adults using the internet for health-seeking purposes, e-mental health could be an effective approach toward the self-management of stress and emotional issues and improving service accessibility, geographic barriers, and anonymity [19].

The development and implementation of e-mental health programs for the prevention of MDEs need to be examined through a sex and age lens. The prevalence of MDEs in women is about two times higher than that in men [26]; however, the Canadian national data show that in 2009, 76% of the total number of suicides were committed by men [27]. Due to the existence of gender norms and the desirability of perceived masculinity, men are less likely than women to seek help and to disclose depressive symptoms and often delay seeking help until symptoms become severe [28,29]. Men are socialized to be emotionally stoic and exemplify traditional masculine characteristics such as independence, self-reliance, and dominance [30]. Men are concerned over the perceived negative judgments from family and friends if they access treatment for depression. Furthermore, it has been found that being male significantly increases the risk of treatment dropout for self-guided Web-based interventions for depression, highlighting differences in treatment compliance and willingness to complete Web-based interventions [31]. These sex-related help-seeking behaviors and social norms may influence men’s and women’s preference for the content and design features of e-mental health programs and the likely use of the program to deal with work-related stress. The use of eHealth programs may also be related to age. For instance, some studies have demonstrated that young adults are more likely to use eHealth programs than the elderly [32], and other studies have shown that younger age is related to low treatment adherence [31]. Given the lack of consensus and knowledge regarding the sex and age differences related to preferred design features and the likely use of e-mental health programs, and treatment adherence, it is imperative to explore design features that may increase their use and thereby, their effectiveness.

In 2015, we initiated a research project called BroMatters, which aimed to develop and evaluate an e-mental health program to be used by working men who are at high risk of having an MDE. To guide the development of the e-mental health program, we conducted a national survey in 2015 among working men who were at high risk of having an MDE, regarding their acceptance and preferred design features of e-mental health programs [33]. In 2016, as a separate study, we repeated this survey in working women who were at high risk of having an MDE. The objective of this analysis was to examine the sex and age differences in the acceptance and preferred design features of the e-mental health program in male and female workers who are at high risk of having an MDE.

## Methods

### Participants

The 2 surveys employed the same methodology as described in a previous publication [33]. The target population for the 2 surveys were individuals who (1) were working at the time of the survey, (2) were aged 18 years or older, (3) did not have an MDE in the past 12 months, (4) had no language barrier to either English or French, and (5) were at high risk of having an MDE based on sex-specific risk prediction algorithms [34]. The random digit dialing method was used to contact household residents across the country. Interviews were conducted by the Bureau d’intervieweurs professionels (BIP) located in Montreal. BIP has access to household telephone and validated cell phone numbers. The household contact was asked to retrieve or provide contact information (eg, the first name) of the household residents who were currently working. If there was more than one potentially eligible individual in the same household, one was randomly selected. Once the participant was fully informed about the objectives and procedures of the study, oral consent and continuation of telephone interview were deemed as adequate consent for participation. Data were collected using computer-assisted telephone interviews, which were completed by trained telephone interviewers in participants’ language of preference (English or French). Data collection occurred over a period of 9 months between March and December 2015. A minimum of 9 callback attempts were spaced over weekdays for the duration of the data collection period. Participants received a Can $20 incentive for each completed interview as a token of appreciation. Detailed call composition for the men’s survey can be found in our previous publication [33]. In the women’s survey, 47,555 phone numbers were called. A majority of the calls (46,300/47,555, 97.36%) were not valid (not in service, fax or modem, answering machine, language barriers, ineligibility, duplications, and refusal before eligibility was assessed). Among 1255 eligible women, 755 (60.1%) were excluded from the study (prolonged absence, incomplete questionnaires, scheduled callbacks not within data collection period, and refusal after eligibility was verified). The data collection for women occurred between January and April 2016. For the 2 surveys, 500 eligible women and 511 eligible men were interviewed. The studies were approved by the Conjoint Health Research Ethics Review Board of the University of Calgary.

### Measurement

The *sex-specific multivariable risk prediction algorithms for major depression* were administered to estimate the risk (probability) of having an MDE in the next 4 years for each participant [34]. The risk prediction models were designed to be used by individuals who did not have an MDE in the past 12 months. On the basis of participant’s exposure to a key set of risk factors (predictors) in the model, the algorithm can generate the absolute risk and probability of having an MDE in the next 4 years, analogous to the Framingham risk prediction algorithm for coronary heart disease [35,36]. The risk prediction algorithms for MDEs were developed and validated using data from 4737 Canadian men and 5864 Canadian women who were aged 18 years or older and who did not have an MDE in the past 12 months [34]. The risk prediction algorithms include age, personal and family history of an MDE, childhood trauma, and ongoing stress and life events. There are also sex-specific predictors in the models (details regarding sex-specific predictors in the models may be found in our previous publication [34]). The predictive power of the risk prediction algorithms was measured by C statistics. The algorithm for men had a C statistic of 0.7953 and the C statistic of the algorithm for women was 0.7667 [34]. The models had excellent calibration with data, as indicated by the Hosmer-Lemeshow test and visual comparison between the predicted and observed risks by decile risk groups [34]. In our study, ≥6.51% and ≥11.19% were defined as high risk for men and women, respectively, which represents the top 2 decile risk groups in the Canadian male and female populations.

*Internet use* was assessed using questions from the 2012 Canadian Internet Use Survey conducted by Statistics Canada, including use of the internet in the past 12 months, number of hours spent on the internet in the past week, use of the internet to search for medical- and health-related information, and perceived importance of the health information on the internet for decision making.

Preferred design features of e-mental health program questions were developed by the BroMatters team members. Participants were asked “We want to hear your opinion about e-mental health programs for dealing with work and stress issues. Electronic health (eHealth) is defined as...For the following features, please indicate how likely it is that you would use them.” For 17 design features, participants were asked how likely they were to use a feature and answered on a 5-point Likert scale ranging from very likely to very unlikely. Open-ended questions were asked about any other features they may want in an e-mental health program, whether the participant and his or her coworkers would use an e-mental health program to deal with work stress, and what makes it difficult to use an e-mental health program. At the end of this set of questions, the participants were asked “would you or your co-workers use an eHealth program to deal with work stress?” The participants answered *yes*, *maybe*, or *no*. For eligible participants, administering the questions and instruments took an average of 22 min to complete.

### Data Analysis

A total of 500 men and 511 women were included in the data analysis. Responses including *do not know* and *refuse to answer* were excluded from the data analysis. There were no missing data for demographic characteristics or general and health-related internet usage. The percentage of missing data (ie, do not know and refuse to answer) for items regarding preferred design features of an e-mental health program ranged from 0% to 2%. Demographic and socioeconomic characteristics of participants were tabulated. The proportions of general and health-related internet usage were compared between women and men using a chi-square test. The proportions of likely use of specific features of e-mental health programs in women were estimated and compared with those in men using chi-square tests. The comparisons were made overall and by age groups. As men and women were compared in 17 specific design features, the Bonferroni correction was used, and the significance level was set at .003.

## Results

### Demographic Characteristics

Table 1 presents the demographic characteristics of the participants included in this study. Compared with men, women were slightly older; were more likely to be divorced, separated, or widowed; and were more likely to have a lower personal annual income, to have a part-time job, and to be from small- or mid-sized organizations. Finally, a higher proportion of women obtained a higher level of education (partial or completed university), as compared with men.

### General and Health-Related Internet Usage

Table 2 presents the general and health-related internet usage for men and women. Men and women did not differ significantly in terms of the time they spent online or the means by which they accessed the internet. For instance, 35.8% (175/489) of men and 38.0% (182/479) of women indicated that within the past 12 months, they had spent less than 5 hours per week online. Similarly, the majority of men (408/489, 83.4%) and women (413/ 479, 86.2%) accessed the internet using various devices (mobile phone, tablet, etc). Both men (256/307, 83.4%) and women (334/408, 81.9%) believed that being able to access health resources on the internet was important or very important. Women (408/479, 85.2%) compared with men (307/489, 62.8%) were significantly more likely to use the internet for medical or health-related information. Conversely, men (231/307, 75.2%) were significantly more likely to state that the internet was useful in helping them make decisions compared with women (277/408, 67.9%).

When participants were asked “would you or your co-workers use an eHealth program to deal with work stress?, women were more likely to report *yes* than men (*P*=.04). Among 511 men, 35.4% (181) reported *yes*, 37.2% (190) reported *maybe*, and 21.9% (112) reported *no*, whereas among 500 women, 42.2% (211) reported *yes*, 34.0% (170) reported *maybe*, and 16.4% (82) reported *no*.

### Preferred Design Features

Table 3 contains the preferred design features in an e-mental health program for men and women. Men and women differed significantly in terms of their e-mental health design feature preferences. Compared with men, a higher proportion of women stated that they were likely to use design features such as practices and exercises to help reduce symptoms of stress and depression (350/495, 70.7%), a self-help interactive program that provides information about stress and work problems (302/489, 61.8%), the ability to ask questions and receive answers from mental health professionals via email or text message (294/491, 59.9%), and to receive printed materials by mail (215/495, 43.4%). Compared with women (104/496, 21.0%), a higher proportion of men (156/509, 30.7%) preferred having some of the program information about ways of dealing with stress and work-related issues to be delivered in a video game format. There were no significant differences between men and women for the remaining design features.

Table 4 presents the preferred design features of an e-mental health program for men and women by age group. Age differences were found in 3 design features in men and women. Compared with older men, men in the youngest age group (36/73, 49%) preferred to receive information on how to deal with work and stress issues in video game format. Men aged between 50 and 64 years (61/140, 43.6%) preferred to receive printed materials by mail rather than having to print online materials oneself, compared with younger men and men over the age of 65 years. Younger men also preferred accessing a program through a smartphone or as an app, with 63% (46/73) of 18- to 29-year olds stating that they would likely use this feature. Younger women also preferred accessing a program through a smartphone or as an app (*P*<.001), considering that of 489 women, 81% (49/60) of 18 to 29 year olds, 64.2% (163/254) of 30 to 49 year olds, 36% (59/162) of 50 to 64 year olds, and 15% (2/13) of women aged 65 years and older said that they were likely to access a program in this manner. There were no other significant age differences in men or women regarding e-mental health design feature preferences.

Sensitivity analyses were conducted in participants who reported likely use of an e-mental health program to deal with work stress and in those with a history of an MDE. In participants who reported likely use of an e-mental health program, information delivered in a video game format and receiving printed materials by mail were still preferred among men (69/179, 38.0%) and women (115/210, 54.8%), respectively. In addition, men preferred to receive information about anger management (98/180, 55%) and women preferred to chart and track mood (133/209, 63.6%). There were no significant differences between men and women for the remaining design features. In participants with a history of an MDE, women were more likely to use a self-help interactive program (139/218, 63.8%), to receive information about improving sleep hygiene (153/216, 70.8%), and to receive printed materials by mail (91/216, 42.1%), and men were more likely to receive information in a video game format (43/130, 33.1%).

**Table 1:** Demographic and socioeconomic characteristics of the participants who were at high risk of major depression classified by sex.

Variable		Men (n=511)	Women (n=500)	*P* value
Age (years), mean (SD)		42.0 (12.2)	44.3 (15.0)	.002
**Marital status, n (%)**				**<.001**
	Married or common-law	389 (76.1)	342 (69.0)	
	Divorced, separated, or widowed	22 (4.3)	68 (13.7)	
	Single	100 (19.6)	86 (17.3)	
**Personal income, n (%)**				**<.001**
	<Can $30,000	60 (12.1)	130 (27.3)	
	Can $30,000-<$60,000	152 (30.6)	216 (45.4)	
	Can $60,000-<$80,000	98 (19.7)	69 (14.5)	
	Can $80,000+	187 (37.6)	61 (12.8)	
**Education level, n (%)**				**<.001**
	<High school	43 (8.4)	12 (2.4)	
	High school	107 (20.9)	81 (16.3)	
	College	167 (32.7)	188 (37.7)	
	University or higher	194 (38.0)	217 (43.6)	
**Employment, n (%)**				**.98**
	Employee	413 (81.5)	396 (81.0)	
	Self-employed	92 (18.2)	91 (18.6)	
	Family business no pay	2 (0.4)	2 (0.4)	
**Job type, n (%)**				**<.001**
	Full time	434 (84.9)	340 (68.0)	
	Part time	37 (7.2)	130 (26.0)	
	Seasonal	18 (3.5)	10 (2.0)	
	Contract	19 (3.7)	18 (3.6)	
	Other	3 (0.6)	2 (0.4)	
**Size of company or worksite, n (%)**				**.01**
	<50	276 (54.2)	290 (59.7)	
	50-499	149 (29.3)	148 (30.4)	
	>500	84 (16.5)	48 (9.9)	
**Provinces, n (%)**				**.16**
	British Columbia	33 (7.1)	58 (13.2)	
	Alberta	52 (11.2)	51 (11.6)	
	Saskatchewan	18 (3.9)	15 (3.4)	
	Manitoba	20 (4.3)	15 (3.4)	
	Ontario	170 (36.6)	161 (36.7)	
	Quebec	141 (30.4)	109 (24.8)	
	New Brunswick	12 (2.6)	8 (1.8)	
	Nova Scotia	12 (2.6)	13 (2.9)	
	Newfoundland	4 (0.9)	7 (1.6)	
	Prince Edward Island	2 (0.4)	2 (0.5)	
**Language, n (%)**				**.31**
	English	374 (73.2)	380 (76.0)	
	French	137 (26.8)	120 (24.0)	
**Work function impairment, n (%)**				**.91**
	None	314 (62.9)	296 (61.4)	
	Mild	146 (29.3)	150 (31.1)	
	Moderate	36 (7.2)	34 (7.1)	
	Severe	3 (0.6)	2 (0.4)	

**Table 2:** General and health-related internet usage during the past 12 months in men and women who were at high risk of major depression.

Internet use		Men		Women		*P* value
		n (%)	95% CI	n (%)	95% CI	
Use internet for personal use (n=1011)		489 (95.7)	93.5-97.2	479 (95.8)	93.6-97.2	.93
**Hours of internet use each week (n=968)**						**.35**
	<5 hours	175 (35.8)	31.6-40.2	182 (38.0)	33.7-42.4	
	5-9 hours	137 (28.0)	24.2-32.2	144 (30.1)	26.1-34.3	
	10-19 hours	113 (23.1)	19.6-27.1	102 (21.3)	17.8-25.2	
	20-29 hours	39 (8.0)	5.9-10.7	35 (7.3)	5.3-10.1	
	30-39 hours	11 (2.3)	1.2-4.0	8 (1.7)	0.8-3.3	
	>40 hours	14 (2.9)	1.7-4.8	6 (1.3)	0.6-2.8	
	Do not know	0 (0)	0-0	2 (0.42)	0.1-1.7	
Access internet with a smart phone, tablet, or other device (n=968)		408 (83.4)	79.9-86.5	413 8(6.2)	82.8-89.0	.23
Used internet for medical or health-related information (n=968)		307 (62.8)	58.4-67.0	408 (85.2)	82.1-88.4	<.001
**How useful was the internet in helping you make a decision (n=715)**						**.04**
	Not useful	32 (10.4)	7.5-14.4	41 (10.1)	7.5-13.4	
	Unsure	41 (13.4)	10.0-17.7	88 (21.6)	17.8-25.8	
	Useful	231 (75.2)	70.1-79.8	277 (67.9)	63.2-72.3	
**How important is it for you to be able to access health resources on the internet (n=715)**						**.22**
	Not important	31 (10.1)	7.2-14.0	32 (7.8)	5.6-10.9	
	Unsure	20 (6.5)	4.2-9.9	41 (10.1)	7.5-13.4	
	Important	256 (83.4)	78.8-87.2	334 (81.9)	78.0-85.3	

**Table 3:** Proportions of preferred design features of e-mental health program in men and women who were at high risk of major depression.

Feature	Men		Women		*P* value
	n (%)	95% CI	n (%)	95% CI	
Practice and exercise to reduce stress	303 (59.5)	55.2-63.7	350 (70.7)	66.5-74.6	<.001
Information about improving sleep hygiene	313 (61.3)	56.9-65.4	329 (66.9)	62.6-70.9	.09
Educational materials	295 (57.8)	53.5-62.1	313 (63.8)	59.4-67.9	.002
Self-help interactive program	244 (47.8)	43.5-52.2	302 (61.8)	57.4-66.0	<.001
Setting personal goals and tracking them	277 (54.6)	50.3-58.9	296 (59.9)	55.5-64.2	.02
Ask questions and receive answers from mental health professionals	253 (49.7)	45.4-54.1	294 (59.9)	55.5-64.1	.005
Direct referral to health professional to deal with work and stress issues in person	263 (51.7)	47.3-56.0	292 (59.5)	55.0-63.7	.004
Being able to access a program via a smartphone or as an app	264 (52.0)	47.6-56.3	273 (55.8)	51.4-60.2	.39
Watching videos online on how to deal with work and stress issues	272 (53.3)	49.0-57.6	271 (54.5)	50.1-58.9	.58
Receiving printed materials by mail	167 (32.7)	28.7-36.9	215 (43.4)	39.1-47.9	<.001
Chart and track mood	217 (42.6)	38.4-47.0	237 (48.1)	43.7-52.5	.15
Access by phone to a trained coach to help with work stress	211 (41.3)	37.1-45.6	235 (48.1)	43.6-52.5	.02
Information about anger management	211 (41.5)	37.2-45.8	190 (38.3)	34.1-42.7	.17
A risk calculator predicting future risk of having major depression	215 (42.6)	38.3-46.9	184 (38.1)	33.9-42.5	.13
Online chat room	129 (25.3)	21.7-29.3	124 (25.0)	21.3-29.0	.25
Online peer connection	140 (27.5)	23.7-31.5	136 (27.8)	24.0-31.9	.02
Information delivered in a video game format	156 (30.7)	26.8-34.8	104 (21.0)	17.6-24.8	.002

**Table 4:** Proportions of preferred design features of e-mental health program in high-risk men by age groups.

Feature	18-29 years		30-49 years		50-64 years		>65 years		*P* value
	n (%)	95% CI	n (%)	95% CI	n (%)	95% CI	n (%)	95% CI	
Information delivered in video game format (n=509)	250 (49.3)	37.8-60.9	151 (29.7)	24.6-35.3	120 (23.7)	17.3-31.6	108 (21.4)	6.0-54.0	.001
Print materials by mail (n=511)	105 (20.6)	12.6-31.6	159 (31.3)	26.2-37.0	222 (43.6)	35.5-52.0	73 (14.3)	2.9-48.0	.001
Access program via smartphone or app (n=508)	320 (63.0)	51.2-73.5	281 (55.3)	49.4-61.1	211 (41.7)	33.7-50.2	145 (28.6)	9.6-60.1	.003

## Discussion

### Principal Findings

The data from the 2 national surveys demonstrate that men and women who were at high risk of developing depression did not differ in terms of their general internet usage. However, women were more likely to use the internet for health- and mental health–related information and demonstrated greater acceptability of the use of e-mental health programs for work stress. Although there were similarities among men and women in terms of preferred e-mental health design features, important sex differences emerged. Women preferred design features that were interactive. The only design feature that was preferred by men, as compared with women, was receiving information in a video game format. Age differences in preferred e-mental health design features were minimal, irrespective of sex.

There were some broad similarities among men and women with respect to design feature preference. Men and women differed significantly in their preference for only 5 of the 17 design features. Information regarding sleep hygiene, information about work stress, and practice and exercise to reduce stress were the top 3 features endorsed by men and women. It is unsurprising that design features, which function to provide health information, were preferred among men and women. Previous research demonstrates that seeking Web-based health information is highly prevalent among internet users [37] and obtaining information is a primary motive for internet use [38].

Notable differences in e-mental health design feature preference were found between men and women. Compared with men, women preferred 5 design features for an e-mental health program including practice and exercise to reduce stress, quality information about work stress, self-help interactive programs, being able to ask questions and receive answers, and receiving printed materials. Men preferred only 1 feature as compared with women—being able to receive information in a game format. These results were replicated in sensitivity analyses among men and women with a history of MDE; men were more likely to use a feature that delivered information in a video game format, whereas women preferred to use a self-help interactive program.

Our findings are in accordance with previous studies, which demonstrate that men and women do not differ in their self-reported general internet usage [39], although women are more likely to use the internet for health-related information [40,41], as compared with men. Previous studies have demonstrated greater acceptability, attitudes, help-seeking, and use of e-mental health programs [30,42] as well as more favorable attitudes toward seeking psychological professional help through face-face, email interaction, and Web-based counseling [43] among women. This study likewise found that women demonstrated greater acceptability of the use of e-mental health programs for work stress.

### Comparison and Interpretation

To our knowledge, no studies have examined sex or age differences in specific e-mental health design feature preference. Thus, direct comparisons with previous studies on sex and age differences in preferred design features are not possible. However, previous research examining health-related internet use has demonstrated that women are more likely to be interactive Web-based health users [40], which is consistent with the results of this study. In comparison with women, men preferred to receive information in a video game format, a finding that is consistent with general video game use across genders, as studies show that men play twice as many video games per week as women [44].

These differences may be useful to consider when designing effective eHealth interventions for mental health. For example, low adherence is a significant problem for most eHealth programs, and this issue may be exacerbated among population groups who are at risk for MDEs but are not currently experiencing an episode [1]. The incorporation of interactive features or gamification strategies to increase adherence may be a useful method of increasing adherence among the overall target audience. Gamification may be especially useful for increasing adherence in men, who are receptive to receiving information in a video game format but also face substantial barriers to seeking mental health treatment [45,46].

A number of factors likely contribute to the observed sex differences in patterns of health information seeking, eHealth acceptability, and design feature preference for an e-mental health program. Compared with men, women may be more likely to search for Web-based mental health information because they have stronger positive beliefs regarding the benefits of Web-based health searches [47] and because it is an efficient method of searching [48]. According to some authors, this can be explained from a social role perspective [49-51]. As women often have a multitasking agenda, especially in middle age, efficiency and convenience may be more highly regarded. Fewer men reported that they would use an eHealth program to manage work-related stress. This reflects the tendency for men to be less likely than women to seek either formal or informal help for mental health issues [30]. As some authors have noted, study participants with a history of seeking Web-based health information have overcome some practical and stigma-related barriers in seeking Web-based psychological help [52]. As such, greater acceptability of e-mental health programs among women may be partially attributed to their greater use of the internet for health and mental health information. Women also tend to have greater psychological openness, which describes the ability to acknowledge a psychological problem and a need for help. Conversely, men have more difficulty identifying feelings of distress as emotional problems [53]. In addition, a number of perceived barriers, such as internalized and treatment stigma [45], masculine norms, communication barriers, the role of self-help strategies, and perceptions of mental health, have been shown to prevent men from accessing Web-based health resources [46]. Other barriers, such as privacy; ease of navigation; personal relevance; and lack of personal interaction, time, and knowledge, may prevent men from using eHealth programs for depression [33]. Thus, there are probable barriers other than attitudes toward help seeking that inhibit men from seeking Web-based help.

The majority of design features preferred among women were interactive in nature. Furthermore, women were more likely to use a guided feature in which they could ask questions and receive answers from a mental health professional. This reflects women’s use of eHealth programs for social motives and enjoyment, in addition to information seeking [48], and their willingness to speak to a mental health professional [43]. Men preferred to receive information in a video game format, compared with women. Men enjoy gaming and seek out game-play for social situations more so than women [54]; consequently, they may find eHealth information more interesting and engaging in this format.

Among men and women who were likely to use an e-mental health program for work stress, few differences in design feature preference were evident. Most notably, women no longer preferred interactive features as compared with men. Men who are likely to use an e-mental health program may have greater acceptability of eHealth programs, resulting in a greater likelihood of using a variety of e-mental health features. Factors such as perceived internet skill and perceived credibility of Web-based information predict the use of Web-based interactive features and may also differ among men who are likely to use an e-mental health program for work stress [55].

To our knowledge, there are no studies that have examined specific design feature preferences of e-mental health programs across age; however, the general use of mobile phones and apps is consistent with our findings: younger individuals are more likely to own a mobile phone [56] and to download and use health-related apps [57] than older individuals. Accordingly, tailoring eHealth interventions to participants’ age may improve the efficacy of eHealth interventions.

### Limitations

First, the findings from this study are limited by the survey data’s reliance on self-reporting. As such, reporting and recall biases are possible and causal inferences cannot be drawn. Second, the surveys in men and in women were not conducted during the same time. The impact of timing of the survey on the results is not clear. Third, both surveys were conducted in Canada; thus, precautions need to be taken when extrapolating the results to other regions. Fourth, the response rate for women was relatively low, as compared with men. Among 1255 eligible women, 755 (60.1%) were excluded from the study because of prolonged absence, incomplete questionnaires, scheduled callbacks not within data collection period, and refusal after eligibility was verified. This may have reflected systematic bias, wherein females with negative attitudes toward psychological treatment or e-mental health have declined to respond to the survey. Furthermore, income level was used to calculate the risk of depression in women; thus, women with a lower income level may be overrepresented in this sample. Fifth, women showed greater acceptability toward the use of e-mental health programs, which could influence design feature preference. Likewise, previous treatment experience was not examined in this study and may have an effect on e-mental health acceptability [58] as well as design feature preference. Sixth, design feature preference was measured by the use of a single item. The use of more items may have generated a score that is more reliable. Finally, although studies have found that treatment acceptability is important to consider as it may improve both treatment adherence [59] and overall outcome [60], and although intention is believed to be the best proximal predictor of actual behavior, it may not translate directly to the actual use of internet technology use [61].

### Conclusions

E-mental health programs can play an important role in the prevention of workplace depression. To enhance the program’s acceptance, adherence, and effectiveness, researchers and program developers should account for patients’ preferences and needs in the design process, including any sex- or age-based differences. This research found that both men and women highly endorsed an intervention containing quality information about stress reduction, work-related stress, and sleep hygiene. However, sex differences emerged. Most notably, women preferred interactive programs that incorporated exercises to reduce stress and Web-based guidance, whereas men preferred receiving information in a video game format. Tailored programs, such as those that involve interactive exercises and guidance or are presented in a video game format, could be beneficial in enhancing e-mental health use in women and men, respectively. Future studies should evaluate the effectiveness of e-mental health programs from sex and age perspectives and identify innovative ways for enhancing the use of e-mental health programs in working men and women at high risk of developing depression.

## Abbreviations

BIP: Bureau d’intervieweurs professionels
CBT: cognitive behavioral therapy
eHealth: electronic health
e-mental health: electronic mental health
MDE: major depressive episode
